# Methylenetetrahydrofolate reductase (MTHFR) C677T and A1298C polymorphisms in breast cancer: a Sardinian preliminary case-control study

**DOI:** 10.7150/ijms.32162

**Published:** 2019-07-22

**Authors:** Paolo Castiglia, Valeria Sanna, Antonio Azara, Maria R. De Miglio, Luciano Murgia, Giovanna Pira, Francesca Sanges, Alessandro Fancellu, Ciriaco Carru, Marco Bisail, Maria Rosaria Muroni

**Affiliations:** 1 Department of Medical, Surgical and Experimental Sciences, University of Sassari, Via P. Manzella, 4 - 07100 Sassari, Italy.; 2 Division of Medical Oncology, AOU Sassari, Via E. De Nicola - 07100 Sassari, Italy; Sassari, Italy.; 3 Department of Biomedical Sciences, University of Sassari, Viale San Pietro 43 - 07100 Sassari, Italy.; 4 LILT, Sassari, Via Amendola, 40/L - 07100 Sassari, Italy.

**Keywords:** Methylenetetrahydrofolate reductase (MTHFR), Folate, Polymorphisms, SNPs C677T and A1298C, Breast cancer.

## Abstract

Two common polymorphisms in the MTHFR gene, C677T and A1298C, are associated with reduced enzyme activity and may be associated with breast cancer susceptibility.

We performed a case-control study to investigate the association between the two SNPs in the MTHFR gene and risk of breast cancer. In total, 58 breast cancer patients and 58 unaffected controls were enrolled in the study. Polymerase chain reaction/restriction fragment length polymorphism technique (PCR-RFLP) was conducted to determine the genotypes.

No significant differences were found in the genotypes of the two polymorphisms of the MTHFR gene between cases and controls. The OR and 95% CI for the 677CC, 677CT and 677TT genotypes were 1.00, 0.95 (0.39-2.31) and 0.87 (0.27-2.80), respectively; those of the 1298AA, 1298AC and 1298CC genotypes were 1.00, 0.59 (0.26-1.36) and 0.78 (1.32-4.66) respectively. Furthermore, it has been shown in patients with breast cancer a risk of presenting with an aggressive biophenotype about twice or three times higher in the presence of the C677T and A1298C polymorphisms, respectively. Finally, the A1298Cpolymorphism is significantly associated with increased recurrence risk of lymph node-positive breast cancer.

Our study has not shown a significant association between MTHFR gene polymorphisms and breast cancer risk. However, it highlighted the key-role played by the presence of mutant alleles for both polymorphisms in increasing the risk of developing more aggressive phenotypes; moreover, specifically in A1298C, it might also lead to a higher risk of developing lymph node metastasis.

## Introduction

The Methyleletetrahydrofolate reductase (MTHFR) gene is located on chromosome 1, it has 11 exons 1 and as shown by quantitative and qualitative studies, its expression is tissue-specific 2. The MTHFR enzyme plays a central role in intracellular folate homeostasis; it is in fact a key enzyme in the folate metabolic pathway and it catalyses the conversion of 5,10-methylenetetrahydrofolate to 5-methyltetrahydrofolate which is the carbon donor for homocysteine methylation to methionine. Subsequently, methionine is converted into S-adenosyl methionine (SAM) which represents the methyl donor in biological processes, including the methylation of proteins and nucleic acids 3. Furthermore, MTHFR plays an essential role in de novo synthesis of purines and the pyrimidine nucleoside hence in DNA biosynthesis, repair and maintenance of DNA stability.

Two functional and well characterized SNPs in the MTHFR gene, C677T (rs 1801133) and A1298C (rs 1801131), have been associated with decreased enzyme activity and increased levels of plasma homocysteine 4,5. In the MTHFR gene, the C677T polymorphism occurs in exon 4, which involves a C to T substitution at position 677, a consequence of transformation from an alanine to a valine at codon 222 in the N-terminal catalytic domain. Individuals with the 677TT homozygous variant have no more than 30% of normal enzyme activity, and heterozygotes CT genotype have 65% of normal enzyme activity and increased thermolability 6,7. An additional polymorphism, MTHFR A1298C, occurs in exon 7 resulting into the change from a glutamic acid to alanine residue at codon 429 in the C-terminal regulatory domain of the protein and decreases the enzyme activity 8. The heterozygous (AC) and homozygous (CC) carriers have a reduction of the enzymatic activity of 15% and 30%, respectively. In individuals with reduced MTHFR enzyme activity, DNA hypomethylation in leukocytes is associated with cardiovascular disease, kidney failure, chromosome anomaly, spontaneous abortion and other diseases 9-11.

The present study aims firstly at evaluating the role of C677T and A1298C polymorphisms as risk factors in a small cohort of healthy women and breast cancer patients. In the literature, study findings on such polymorphisms are discordant, due to the fact that they are strongly influenced by differences among specific ethnic groups. For what concerns the Sardinian area, these polymorphisms have never been studied: thus regardless the results found, a higher number of cases will have to be analysed in the future. Secondly, the study aims at associating data obtained by molecular subtypes from diagnosed tumours to related clinical parameters in order to evaluate the potential role of the two polymorphisms, then using the latter as prognostic and predictive factors in breast cancer disease development.

## Materials and Methods

### Subjects of Study

A total of 116 women were evaluated in this case-control study: 58 breast cancer patients enrolled at the Medical Oncology Unit, Sassari University Hospital, Sassari and 58 healthy women without history of cancer enrolled by the local branch of Lega Italiana Lotta contro i Tumori (LILT), Sassari (Italy).

All women taking part to the study were asked to fill in a questionnaire regarding all known risk factors for breast cancer development such as age, body weight, reproductive-hormone profile, cancer familiarity, smoking habits, alcohol, lifestyle and diet. The questionnaire was administered to healthy women at Sassari LILT local office, while women affected by breast cancer were interviewed at the Medical Oncology Unit, Sassari University Hospital.

In addition, venous blood samples were collected from both patient's groups.

Each part of the questionnaire has been accurately and thoroughly explained during the meetings held to enrol healthy women so as to transform these meetings into learning opportunities aimed at prevention; probably due also to this approach, a study adherence over 90% has been achieved.

On the other hand, the cancer patients enrolled were diagnosed with infiltrating ductal carcinoma and some cases presented with ductal carcinoma * in situ*, with age between 36 to 85 years. The overall adherence to the study has been approximately 75%.

This experimental study was approved by ASL Sassari Bioethical Committee and written informed consent was obtained from each participant, according to Italian legislation.

### MTHFR Genotyping

The MTHFR polymorphisms were analyzed by a polymerase chain reaction restriction fragment length polymorphism (PCR-RFLP) assay. For each analyzed polymorphism, 10% of the total samples were randomly retested with 100% concordance.

All participants were requested to provide 3 ml peripheral whole blood, which was collected in K3-EDTA, aliquoted and stored at -20°C.

Genomic DNA was isolated from 200 μl of whole blood, using QIAmp DNA Blood Mini Kit (Qiagen, Germany) following manufacturer's instructions.

*MTHFR C677T*. PCR was performed with a final volume of 20 μl of reaction mixture containing 100 ng DNA, 1x PCR buffer, 1,5 mM of MgCl2, 0,2 mM of each dNTP, 1,25 units of Taq Gold DNA polymerase (Applied Biosystems, USA) and 0,6 mM of each primer: forward 5I-TGAAGGAGAAGGTGTCTGCGGGA-3I and reverse primer 3I-AGGACGGTGCGGTGAGAGTG-5I, yielding a 201-bp fragment 7. PCR conditions consisted of an initial denaturation at 95°C for 5 minutes, followed by 35 cycles of 95°C for 30 s, 60°C for 30 s, 72°C for 30 s with a final extension at 72°C for 5 minutes.

**Figure 1:**
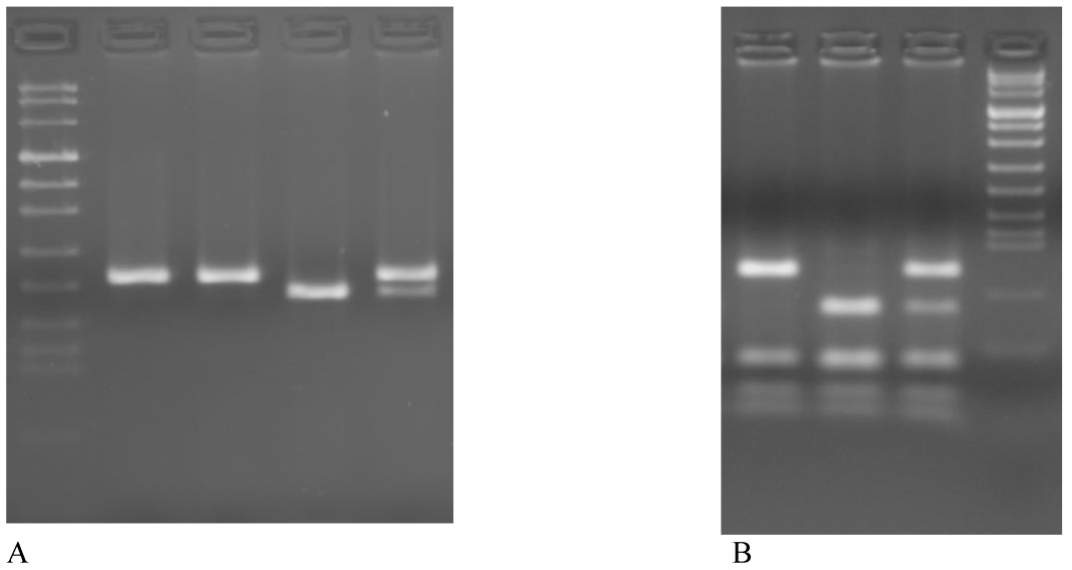
Representative examples of genotyping of MTHFR polymorphism on 3% agarose gel. **A** *MTHFR C677T*: lane 1 Marker VIII, lane 1-2 CC (201bp), lane 3 TT (178bp + 23bp), lane 4 CT (201bp + 178bp + 23bp). **B** *MTHFR A1298C:* lane 1 CC (84bp + 31bp + 30bp + 28bp + 18bp), lane 2 AA (56bp + 31bp + 30bp + 28bp + 18bp), lane 3 AC (84bp + 56bp + 31bp + 30bp + 28bp + 18bp), lane 4 Marker VIII

The PCR products were digested with HinfI (New England Biolabs, USA) in a volume of 20μl for 3 hours at 37°C, electrophoresed in a 3% Metaphor Agarose gel and visualized by ethidium bromide staining. The C→T valine variant creates a HinfI restriction enzyme site and produces fragments of 178-bp and 23-bp (Figure 1A).

*MTHFR A1298C*. This polymorphism consists of a substitution from A to C in codon 429 (gaa→gca) of exon 7 of the gene and converts a glutamic acid into alanine, cancelling the cutting site for the enzyme MboII. PCR was performed with the final volume of 25 μl of reaction mixture containing 150 ng DNA, 5x PCR buffer, 0,2 mM of each dNTP, 2,5 units of Q5 Hot Start High-Fidelity DNA Polymerase (New England Biolabs, USA) and 0,6 mM of each primer: forward 5I-CTTTGGGGAGCTGAAGGACTACTAC-3I and reverse primer 3I-CACTTTGTGACCATTCCGGTTTG-5I, yielding a 163-bp fragment 12. PCR conditions consisted of an initial denaturation at 98°C for 30 s, followed by 35 cycles of 98°C for 30 s, 60°C for 30 s, 72°C for 30s with a final extension at 72°C for 5 minutes.

The PCR products were digested with MboII (New England Biolabs, USA) in a volume of 25 μl for 3h at 37°C, electrophoresed in a 3% Metaphor agarose gel and visualized by ethidium bromide staining. The 1298C allele abolishing one of the MboII restriction site, therefore digestion of the 163 bp PCR product of the 1298A allele yields 5 fragment of 56, 31, 30, 28 and 18 bp, whereas the 1298C allele resulting in 4 PCR bands of 84, 31, 30 and 18 bp (Figure 1B).

### Statistical Analysis

For what concerns the descriptive statistics analysis of continuous quantity variables, the mean and standard deviation were calculated, as well as the median and interquartile range, when appropriate. In relation to quantity variables, the percent distribution was described, while for dichotomous variables the prevalence was calculated. As far as the inferential analysis is concerned, the process of assessing the prevalence and the differences between prevalences observed was conducted by using the Z-test. Differences between mean values of Gaussian distribution variables or whose numerosity was sufficiently consistent, were calculated by a two-tailed Student's t-test on independent samples. The association strength between determinants and dichotomous outcome has been evaluated through Odds ratio. On the whole, inferential analysis were performed with an error of first type α ≤ 0,05. Where appropriate, the 95% confidence interval estimate was used. The 95% OR confidence intervals were calculated by using the Woolf method for 2x2 tables or through unconditional logistic regression for multivariate analysis.

## Results

### Demographic characteristics

The composition of the two analysed samples and data related to the most important variables analysed are shown in Table 1. A statistically significant difference can be noticed between the two groups for what concerns age, Body Mass Index (BMI), *nulliparae* prevalence and taking oral contraceptives. In fact, it can be seen that the age of cancer patients is higher (55,7 ± 10,4) than healthy controls (49,2 ± 14,7), their BMI is also higher (25,1 ± 4,4 vs 22,3 ± 4,98), a lower number of *nulliparae* (20,7% vs 37,9%) and a reduced use of oral contraceptives (60,3% vs 82,8%). No statistically significant differences are seen between the two groups for what concerns the onset of menstruation, familiarity of breast cancer and cigarettes and alcohol consumption.

### MTHFR polymorphisms and susceptibility to BC

For what concerns epidemiological and molecular analysis results, Table 2 shows MTHFR C677T and MTHFR A1298C polymorphism distribution in 58 women for the breast cancer cohort and in 58 controls. Ten cases (17,24%) and eleven (18,97%) controls were homozygous for the C677T variant allele. Taking into consideration together the CT heterozygous and the TT homozygous genotypes, it can be seen a non-significant risk reduction compared to the wild-type CC genotype (OR= 0,93, CI 0,41-2,11). For what concerns the A1298C polymorphism, four cases among the breast cancer patients (6,9%) and four in controls (6,9%) were homozygous for the CC variant allele. Likewise, in genotypes AC and CC together the OR was lower compared to the AA wild-type genotype. Besides, data related to the combined genotypes of the two MTHFR gene polymorphisms are reported.

### Association between polymorphisms and clinical data

Among patients enrolled in our study, it can be observed that the most recurrent molecular biophenotype (data not shown) is Luminal A (36,84%), followed by * in situ* tumour (15,79%) and by Luminal B Her2 negative (15,79%). Less recurrent are those characterized by more aggressive biophenotypes (Luminal B Her2 positive 14,04%, Her2+ non luminal 7,02% and triple negative 10,53%).

Moreover, an association between each polymorphism and variable considered important for prognostic and/or predictive purposes has been analysed. The association with the biophenotype is reported in Table 3: more specifically with MTHFR A1298C in Table 3a and with MTHFR C677T in Table 3b. In general, it can be noticed a tendency towards association between aggressive biotypes and the mutant allele variants in the two MTHFR polymorphisms, leading to a three-fold increased risk of having an aggressive biophenotype in the case of MTHFR A1298C polymorphism and a two-fold risk increase for MTHFR C677T polymorphism.

Cases with metastasis were 46% of the total number of breast cancer. The risk of lymph node metastasis resulted significantly increased of 2,5 times for each mutant allele in A1298C polymorphism (Table 4). No variation has instead been observed in C677T.

**Figure 2:**
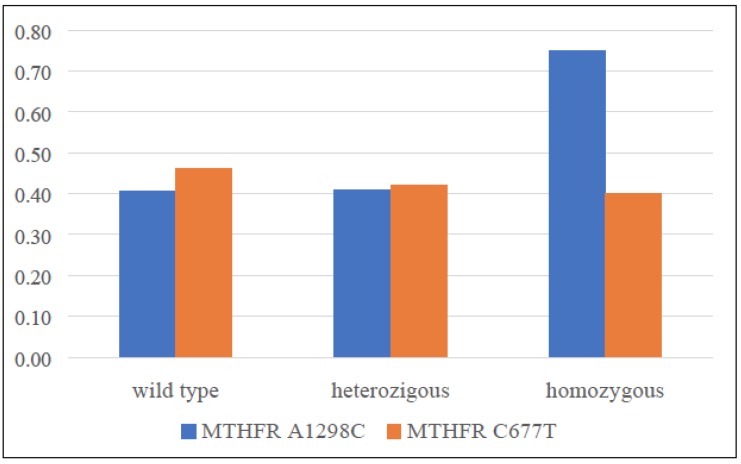
Patients overweight prevalence distribution in relation to the two analysed polymorphisms.

In conclusion, a significant increase of overweight (BMI>25) prevalence among patients with a CC homozygous mutant allele of A1298C polymorphism has been observed (Figure 2).

## Discussion

Breast cancer is a complex and heterogeneous disease with a multifactorial etiology. Several studies have found an association between the C677T and A1298C polymorphisms and an increase in the risk of various types of cancer including pancreas, esophagus, stomach, colon, lung and, breast cancer 13-17. The risk of tumour development associated with alleles that causes a reduction in the activity of the MTHFR enzyme is influenced by environmental factors such as the proportion of folates introduced with the diet 18. Several evidences suggest an association between 677T and 1298C alleles and susceptibility to breast cancer, 19-26 although divergent results have been found 27-34.

The present study was conducted on the basis of a detailed questionnaire on different known risk factors, including the patient's diet composition. The descriptive analysis on the two compared groups highlighted significant differences concerning some variables such as age, BMI, *nullipara* status and use of oral contraception. Instead, no significant differences were observed for what concerns age of first menstruation, cigarette and alcohol consumption, and breast cancer familiarity. This result, apparently in contrast to what should have been expected, is probably related to the method used to enrol the control group. The latter was in fact formed by women consecutively enrolled who voluntarily accessed the LILT local office for preventive counselling and investigation. Women from any socioeconomic status might access to LILT, but there might be a self-selection bias as women with cancer familiarity are probably more sensitive to the issue; thus they might be more used to undergo periodic medical check-ups at LILT, being more likely to be selected as controls. This consideration might be confirmed by the extremely reduced absence of a difference in tumour familiarity prevalence observed between the two groups.

Based on epidemiological and statistical analysis conducted on the different MTHFR polymorphic patterns between the two examined groups, no relevant data emerged to confirm an association of C677T and A1298C with a higher breast cancer risk. With the aim to shed light on the link between the two aforementioned polymorphisms and the breast cancer etiology in our area, a prosecution of the study has been foreseen, including the collection of a larger number of samples compared to the present study, as well an extra control group enrolled taking into consideration the self-selection bias.

For what concerns the biophenotype of tumours observed in the study cases, the pattern does not differ from what expected based on literature. Therefore, the sample well represents the cancer population subjected to the study. The risk inferences are thus consistent. As a consequence, the result of associating the biophenotype and the polymorphisms highlights the risk of presenting with an aggressive biophenotype which is two or three fold higher in patients with polymorphisms C677T and A1298C, respectively. In relation to the risk of having a lymph node metastasis, the analysis based on unconditional logistic regression shows that the A1298C polymorphism results highly and strongly associated to such a risk, showing a positivity that is likely to increase approximately 2,5 times in each mutant allele. For what concerns other variables potentially associated to breast cancer risk, the present study shows an association between overweight and homozygous CC mutant allele of A1298C polymorphism.

Based on this observation and discussion, to the best of our knowledge we can declare that the current study represents the first observational analysis on the association between breast cancer and C677T and A1298C polymorphisms of MTHFR gene in Northern Sardinian female population.

The study can be considered the pathfinder for a wider research project and preliminary results here reported strongly indicate the need to carry on the research through increasing the number of subjects analysed in both groups. Increasing the sample and including a second control group will lead to higher effectiveness so as to analyse potential associations and or interactions with other behavioural variables emerging from the questionnaire.

In conclusion, although our study has not ultimately confirmed the association between MTHFR gene polymorphism and breast cancer risk, it clearly shows the crucial key-role of mutant allele presence of both polymorphisms in relation to the risk of developing more aggressive biophenotypes and, just in the case of A1298C, an increased risk to develop lymph node metastasis.

The biological plausibility of the association of MTHFR polymorphisms with the risk of developing more aggressive biophenotypes is supported by numerous evidences that the deregulation of DNA methylation, de novo synthesis of purines and pyrimidine nucleoside as well as maintaining DNA integrity by MTFHR polymorphic variants, which determine conformational modifications leading to isoenzymes with altered functional activity, may contribute to malignant transformation 35,36.

However the chance that significant association is really true should be better investigated 37.

Therefore, a higher number of cases analysed might ultimately define the role played by different polymorphisms on cancer risk and might, on one hand, enable to better define the prognosis of observed cases and on the other hand, it might allow the identification of tools for early diagnosis to be stratified on differentiated risk profiles in order to increase the efficacy of screening campaigns.

In conclusion, although with the limitations described, our results suggest a biological role of the MTHFR enzyme and its polymorphisms in the progression of breast cancer, related to the association of MTHFR polymorphisms with aggressive biophenotype and lymph node metastasis. Extensive and independent study should be warranted to evaluate the biological mechanisms of this association.

## Figures and Tables

**Table 1:** Sample distribution based on group (patients and healthy controls) and main study variables.

	Cases Mean value (±DS)	Controls Mean value (±DS)	P t-student test
Age	55,66 ± 10,37	49,17 ± 14,71	0,006
BMI	25,11 ± 4,37	22,28 ± 4,98	0,003
Age of menarche	12,5 ± 1,52	12,5 ± 1,3	0,46
	**%**	**%**	**z-test**
Nulliparae	20,69	37,93	0,0410
Oral Contraceptives	60,34	83	0,0068
Breast cancer familiarity	53,45	52	0,87
Smoking consumption	25,86	21	0,53
Alcohol consumption	62,07	65,52	0,69

**Table 2:** MTHFR C677T and A1298C genotypes in breast cancer patients and controls.

MTHFR	Genotypes and alleles	Patients N (%)	Controls N (%)	OR (95% CI)	P
C677T	CC CT TT CT+TT C T	58 22 (37,93) 26 (44,83) 10 (17,24) 36 (62,07) 70 (60,34) 46 (39,65)	58 21 (36,20) 26 (44,83) 11 (18,97) 37 (63,79) 68 (58,62) 48 (41,37)	1 0.95 (0.39-2.31) 0.87 (0.27-2.80) 0.93 (0.41-2.11) 1 0.93 (0.53-1.63)	0.91 0.79 0.84 0.79
A1298C	AA ACCCAC+CC A C	32 (55,17) 22 (37,93) 4 (6,90) 26 (44,83) 86 (74,14) 30 (25,86)	25 (43,10) 29 (50,0) 4 (6,90) 33 (56,90) 79 (68,10) 37 (31,90)	1 0.59 (0.26-1.36) 0.78 (1.32-4.66) 0.62 (0.28-1.37) 1 0.75 (0.40-1.37)	0.18 0.74 0.19 0.31
Combined Genotypes (677/1298)	CC/AA CC/AC CC/CCCT/AACT/ACCT/CC TT/AATT/ACTT/CC	6 (10,34) 12 (20,69) 4 (6,90) 17 (29,31) 9 (15,52) 010 (17,24) 00	3 (5,17) 14 (24,14) 4 (6,90) 11 (18,96) 15 (25,86) 011 (18,97) 00	1 0.43 (0.58-2.61) 0.50 (0.05-5.30) 0.77 (0.10-4.63) 0.30 (0.04-1.90) -- 0.46 (0.06-2.94) -- --	0.29 0.49 0.75 0.13 -- 0.34 -- --

**Table 3:** Association between MTHFR A1298C and C677T polymorphisms and molecular biophenotype.

**a) Association between biophenotype and MTHFR A1298C**				
	Wild type (N°)	Mutant (N°)	OR	IC 95%
Aggressive biophenotype	7	11		
Non-aggressive biophenotype	25	14		
Total	32	25	2,80	0,77-10,50
**b) Association between biophenotype and MTHFR C677T**				
	Wild type (N°)	Mutant (N°)	OR	IC 95%
Aggressive biophenotype	5	13		
Non-aggressive biophenotype	17	22		
Total	22	35	2,01	0,52-8,55

**Table 4:** Association between N stage and MTHFR A1298C polymorphism.

	N-	N+	OR	IC 95%
Wild type (AA)	21	11		
Heterozygous (AC)	9	12		
Homozygous (CC)	1	3		
Total	31	26	2,47	1,004-6,096

## References

[1] Goyette P, Samner JS, Milos R (1994). Human methylenethetrahydrofolate reductase: isolation of cDNA, mapping and mutation identification. Nat Genet.

[2] Gaughan DJ, Barbaux S, Kluijtmans LAJ (2000). The human and mouse methylenetetrahydrofolate reductase (MTHFR) genes: genomic organization, mRNA structure and linkage to the CLCN6 gene. Gene.

[3] Liew S, Gupta ED (2015). Methylenethetrahydrofolate reductase (MTHFR) C677T polymorphism: Epidemiology, metabolism and the associated diseases. Eur J Medical Genetics.

[4] Weisberg IS, Jacques PF, Selhud J (2001). The1298A>C polymorphism in methylenetetrahydrofolate reductase (MTHFR): *in vitro* expression and association with homocysteine. Atherosclerosis.

[5] Rozen R (1997). Genetic predisposition to hyperhomocysteinemia: deficiency of methylenetetrahydrofolate reductase (MTHFR). Thromb Haemost.

[6] Van Der Put NM, Gabreels F, Stevens EM, Smeitink JA, Trijbels FJ, Eskes TK, Van den Heuvel LP, Blom HJ (1998). A second common mutation in the methylenetetrahydrofolate reductase gene: an additional risk factor for neural-tube defects?. Am J Hum Genet.

[7] Frosst P, Blom HJ, Milos R, Goyette P, Sheppard CA, Matthews RG, Boers GJ, den Heijer M, Kluijtmans LA, van den Heuvel LP, Rozen RA (1995). Candidate genetic risk factor for vascular desease: a common mutation in methylenetetrahydrofolate reductase. Nature Genet.

[8] Weisberg I, Tran P, Christensen B, Sibani S, Rozen R (1998). A second genetic polymorphism in methylenetetrahydrofolate reductase (MTHFR) associated with decreased enzyme activity. Mol Genet Metab.

[9] Lievers KJA, Boers GHJ, Verhoef P (2001). A second common variant in the methylenetetrahydrofolate reductase (MTHFR) gene and its relationship to MTHFR enzyme activity, homocysteine, and cardiovascular disease risk. J Mol Med.

[10] Kim JW, Park HM, Choi YK (2011) Polymorphisms in genes involved in folate metabolism and plasma DNA methylation in colorectal cancer patients. Oncol Rep 25(1):167-72.

[11] Castro R, Rivera I, Ravasco P (2004). 5,10-methylenetetrahydrofolate reductase (MTHFR) 677 C/T and 1298 A/C mutations are associated with DNA hypomethylation. J Med Genet.

[12] Naghibalhossaini F, Ehyakonandeh H, Nikseresht A, Kamali E (2015). Association between MTHFR genetic variants and multiple sclerosis in a southern Iranian population. Int J Mol Cell Med.

[13] Hosseini M, Houshmand M, Ebrahimi A (2011). MTHFR polymorphisms and breast cancer risk. Arch Med Sci.

[14] Izmirli M (2013). A literature review of MTHFR (C677T and A1298C polymorphisms) and cancer risk. Mol Biol Rep.

[15] Boccia S, Gianfagna F, Persiani R, La Greca A, Arzani D, Rausei S, D'ugo D, Magistrelli P, Villari P, Van Duijn CM, Ricciardi G (2007). Methylenetetrahydrofolate reductase C677T and A1298C polymorphisms and susceptibility to gastric adenocarcinoma in an Italian population. Biomarkers.

[16] Graziano F, Kawakami K, Ruzzo A, Watanabe G, Santini D, Pizzagalli F, Bisonni R, Mari D, Floriani I, Catalano V, Silva R, Tonini G, Torri V, Giustini L, Magnani M (2006). Methylenetetrahydrofolate reductase 677C/T gene polymorphism, gastric cancer susceptibility and genomic DNA hypomethylation in an at-risk Italian population. Int J Cancer.

[17] Boccia S, Hung R, Ricciardi G (2008). Meta- and pooled analyses of the methylenetetrahydrofolate reductase C677T and A1298C polymorphisms and gastric cancer risk: a huge-GSEC review. Am J Epidemiol.

[18] Ma E, Iwasaki M, Junko I (2009). Dietary intake of folate, vitamin B6, and vitamin B12, genetic polymorphism of related enzymes, and risk of breast cancer: a case control study in Brazilian women.

[19] Gao CM, Tang JH, Cao HX (2009). MTHFR polymorphisms, dietary folate intake and breast cancer risk in Chinese women. J Hum Genet.

[20] Chou YC, Wu MH, Yu JC (2006). Genetic polymorphisms of the methylenetetrahydrofolate reductase gene, plasma folate levels and breast cancer susceptibility: a case-control study in Taiwan. Carcinogenesis.

[21] Wang ZG, Cui W, Yang LF, Zhu YQ (2014). Association of dietary intake of folate and MTHFR genotype with breast cancer risk. Genet Mol Res.

[22] He JM, Pu YD, Wu YJ, Qin R (2014). Association between dietary intake of folate and MTHFR and MTR genotye with risk of breast cancer. Genet Mol Res.

[23] Pepe C1, Guidugli L, Sensi E, Aretini P, D'Andrea E, Montagna M, Manoukian S, Ottini L, Radice P, Viel A, Bevilacqua G, Caligo MA (2007). Methyl group metabolism gene polymorphisms as modifier of breast cancer risk in Italian BRCA1/2 carriers. Breast Cancer Res Treat.

[24] Campbell IG, Baxter SW, Eccles DM, Choong DYH (2002). Methylenetetrahydrofolate reductase polymorphism and susceptibility to breast cancer. Breast Cancer Res.

[25] Semenza JC, Delfino RJ, Ziogas A, Anton-Culver H (2003). Breast cancer risk and methylenetetrahydrofolate reductase polymorphism. Breast Cancer Res Treat.

[26] Matejcic M, de Batlle J, Ricci C, Biessy C, Perrier F, Huybrechts I, Weiderpass E, Boutron-Ruault MC, Cadeau C, His M, Cox DG, Boeing H, Fortner RT, Kaaks R10, Lagiou P, Trichopoulou A, Benetou V, Tumino R, Panico S, Sieri S, Palli D, Ricceri F, Bueno-de-Mesquita HB, Skeie G, Amiano P, Sánchez MJ, Chirlaque MD, Barricarte A, Quirós JR, Buckland G, van Gils CH, Peeters PH, Key TJ, Riboli E, Gylling B, Zeleniuch-Jacquotte A, Gunter M, Romieu I, Chajès V (2017). Biomarkers of folate and vitamin B12 and breast cancer risk: report from the EPIC cohort. Int J Cancer.

[27] Lee SA, Kang D, Nishio H, Lee MJ, Kim DH, Han W, Yoo KY, Ahn SH, Choe KJ, Hirvonen A, Noh DY (2004). Methylenetetrahydrofolate reductase polymorphism, diet, and breast cancer in Korean women. Exp Mol Med.

[28] Gershoni-Baruch R, Dagan E, Israeli D, Kasinetz L, Kadouri E, Friedman E (2000). Association of the C677T polymorphism in the MTHFR gene with breast and/or ovarian cancer risk in Jewish women. Eur J Cancer.

[29] Sharp L, Little J, Schofield AC, Pavlidou E, Cotton SC, Miedzybrodzka Z, Baird JO, Haites NE, Heys SD, Grubb DA (2002). Folate and breast cancer: the role of polymorphisms in methylenetetrahydrofolate reductase (MTHFR). Cancer Lett.

[30] Bravatà V (2015). Controversial roles of methylenetetrahydrofolate reductase polymorphisms and folate in breast cancer disease. Int J Food Sci Nutr.

[31] Reljic A, Simundic AM, Topic E, Nikolac N, Justinic D, Stefanovic M (2007). The methylenetetrahydrofolate reductase (MTHFR) C677T polymorphism and cancer risk: the Croatian case-control study. Clin Biochem.

[32] Grieu F, Powell B, Beilby J, Iacopetta B (2004). Methylenetetrahydrofolate reductase and thymidylate synthase polymorphisms are not associated with breast cancer risk or phenotype. Anticancer Res.

[33] Mohammadzadeh G, Karimi M, Bazyar M, Hosseini SM (2016). Lack of association between MTHFR C677T polymorphism and breast cancer risk in Ahvaz, west south-Iran. Adv Biomed Res.

[34] Hekim N, Ergen A, Yaylim I, Yilmaz H, Zeybek U, Oztürk O, Isbir T (2007). No association between methylenetetrahydrofolate reductase C677T polymorphism and breast cancer. Cell Biochem Funct.

[35] Choi SW, Mason JB (2000). Folate and carcinogenesis: an integrated scheme. J Nutr.

[36] Duthie SJ (1999). Folic acid deficiency and cancer: mechanisms of DNA instability. Br Med Bull.

[37] Antognelli C, Mezzasoma L, Mearini E, Talesa VN (2013). Glyoxalase 1-419C>A variant is associated with oxidative stress: implications in prostate cancer progression. PLOS ONE.

